# Notes and News

**Published:** 1903-07-11

**Authors:** 


### Notes and News.

The Duke and Duchess of Connaught opened a new ward and out-patients' department at the Leyton, Walthamstow and Wanstead Hospital this week. The ward will accommodate upwards of 35 patients. The out-patients' department includes an operating-room, a casualty-room, and new nurses' quarters.

The International Congress of Dermatology will be held in September next year, at Berlin.

Mr. Long has stated in Parliament that he proposes issuing instructions to Poor Law inspectors to include in their report on workhouses one on the phthisical cases found in each.

Although throughout the country generally small-pox continues to diminish, there has been a severe epidemic at Newstead, in Nottinghamshire, which has spread into the districts around.

The Imperial Vaccination League has issued a paper entitled "Ten Answers to Questions on the Subject of Vaccination, for the Use of Parliamentary Candidates." But we think the paper should be far more generally spread throughout the country. Sanitary inspectors, medical officers of health, School Board teachers, and others find the question constantly arising, and the authority of printed statements would certainly help them.

The London County Council's new epileptic colony at Ewell was opened last week by the Duke and Duchess of Fife. The buildings consist of eight separate villas and an administration block. Three hundred and twenty-six patients are to be received, and they will be housed under the charge of a resident married couple in each villa. Dr. Charles Bond, who has had experience at Berley, Banstead, the National Hospital for Epilepsy, and at the Wakefield and Morningside Asylums, has been elected medical superintendent.

The annual dinner of the Association of British Postal Medical Officers will be held at the Hotel Metropole on July 17.

The Metropolitan Asylums Board sends annually from the training ships to the Navy the majority of all the boys who enter the service from any other training ships, except industrial and reformatory ships. They are now asking the Admiralty to accord another ship to them, in view of the services already rendered.

The French Government is making arrangements to provide lady doctors for the native women in Algeria. Medical women are to be placed in all the largest villages and they are to be paid by the government, the consultations being free to the natives. A dispensary has been opened at Algiers, and others will be opened elsewhere shortly.

The deterioration of the English stamina is being made a parliamentary question, and inspired by the results of the Commission of Enquiry into the Condition of Scottish School Children, it appears probable that an English Commission may be appointed also. But the common-sense view of the whole question is that the reformers must concern themselves first with the dwellers in cities, their crowded and insanitary habitations, habits, and poor food.

An interesting Child-Training Exhibition is to be held in St. Petersburg in November, under the patronage of the Dowager Empress. A congress will be held in connection with the exhibition, and it is to be hoped that England will be well represented, as both in hygiene and educational methods there is much to be learnt from foreign nations, and the impression that it is not so is running England into the danger of being left far behind in the race for progress.

This is the time of year when appeals are being issued by all manner of holiday funds for poor children. The appeal is a popular one, and for the most part the generous public does not stop to consider the comparative merits of the appeals, but they give probably to the first who asks. But, if a little more thinking were expended on the subject, all would agree to the vast superiority of the long holiday as opposed to the "day in the country." This latter, whilst sounding so delightful, often ends in the return of weary and over-excited little ones, who are upset for days after by the outing, and some of the good effect is at any rate discounted. But a fortnight, or even a week, away from slum air and unhealthy homes, and an exchange to fresh breezes and more abundant food, will not only expand the mind of a child, but send it back in better health also. So carefully have the ways and means been studied to carry out a holiday at the smallest cost possible, that for a few shillings a child can now secure the immense advantage of a healthy holiday. The Ragged School Union, for instance, sends numbers of children to the country each summer for a fortnight, for the sum of ten shillings only. We can imagine no better return for the value of ten shillings than this, and we hope that the scheme has but to be better known to receive all the support it calls for.

